# Prevalence and factors affecting use of long acting and permanent contraceptive methods in Jinka town, Southern Ethiopia: a cross sectional study

**DOI:** 10.11604/pamj.2014.18.98.3421

**Published:** 2014-05-27

**Authors:** Getachew Mekonnen, Fikre Enquselassie, Gezahegn Tesfaye, Agumasie Semahegn

**Affiliations:** 1 Gambela Regional Health Bureau, Gambela, Ethiopia; 2 School of Public Health, Addis Ababa University, Addis Ababa, Ethiopia; 3 College of Health and Medical Sciences, Haramaya University, Harar, Ethiopia

**Keywords:** Long acting, permanent, contraceptive, women, Ethiopia

## Abstract

**Introduction:**

In Ethiopia, knowledge of contraceptive methods is high though there is low contraceptive prevalence rate. This study was aimed to assess prevalence and associated factors of long acting and permanent contraceptive methods in Jinka town, southern Ethiopia.

**Methods:**

Community based cross sectional survey was conducted to assess the prevalence and factors affecting long acting and permanent methods of contraceptives utilization from March to April 2008. Eight hundred child bearing age women were participated in the quantitative study and 32 purposively selected focus group discussants were participated in the qualitative study. Face to face interview was used for data collection. Data were analyzed by SPSS version 13.0 statistical software. Descriptive statistics and logistic regression were computed to analyze the data.

**Results:**

The prevalence of long acting and permanent contraceptive method was 7.3%. Three fourth (76.1%) of the women have ever heard about implants and implant 28 (50%) were the most widely used method. Almost two third of women had intention to use long acting and permanent methods. Knowledge of contraceptive and age of women have significant association with the use of long acting and permanent contraceptive methods.

**Conclusion:**

The overall prevalence of long acting and permanent contraceptive method was low. Knowledge of contraceptive and age of women have significant association with use of long acting and permanent contraceptive. Extensive health information should be provided.

## Introduction

An estimated 550,000 women die every year through unsafely induced abortion, pregnancy and childbirth. At least 35% of these died due to pregnancies that would be avoided if contraceptives were available [1]. Contraceptive use and fertility rates vary substantially among developing countries. A few countries of Asia and Latin America, at least three-fourths of married women use a contraceptive method. In contrast, in some Sub-Saharan African countries fewer than 10% of married women use contraception. Fertility rates range from just 2.3 children per woman in Vietnam to 7.2 in Niger in developing countries [2]. According to Federal Democratic Republic of Ethiopia central statistics authority's report, Ethiopia has a population of more than 77 million and out of this Southern Nations Nationalities and people regional state has an estimated total population of 15,321,000 [3]. In Ethiopia, family-planning program was first started in the 1960s by family guidance association of Ethiopia. Among the many sexual and reproductive health problems faced by women in Ethiopia such as gender inequality, sexual coercion, early marriage, polygamy, female genital cutting, closely spaced pregnancies, abortion, sexually transmitted infections and AIDS [4, 5]. In Ethiopia, 34% of married women had an unmet need for family planning, with 20% having an unmet need for spacing and 14% having an unmet need for limiting. In Ethiopia, the contraceptive prevalence rate for married Ethiopian women who are currently using a method of family planning is 15%. Almost all of these users are using modern methods. The most widely used methods were Injectables (10%) and pill (3%) [6]. Lacks of access and quality of family planning service have devastating maternal health consequences. In Ethiopia mother die due to avoidable causes related to pregnancy and birth complications in which maternal mortality was 871/100,000 live births [6]. Birth control methods have significant role on the reduction of maternal health problem and prevention of maternal mortality and also the achievement of millennium development goals. This study was aimed to assess prevalence and associated factors of long acting and permanent contraceptive methods in Jinka town, southern Ethiopia.

## Methods

### Study setting

The study was conducted in Jinka town, South Omo zone, in Southern nations and nationalities and people regional state, Ethiopia from March to April 2008. There were 25804 (male-13160, female-12644) peoples at the time of the survey. South Omo is a zone in the Ethiopian Southern nations, nationalities and peoples region, which is bordered on the south by Kenya, on the southwest by the Ilemi Triangle, on the west by Bench Maji, on the northwest by Keffa, on the north by Konta, Gamo Gofa and Basketo, on the northeast by Dirashe and Konso, and on the east by the Oromia Region. The administrative center of south Omo zone is Jinka.

### Study design and Sampling procedure

A community based cross-sectional study design involving both quantitative and qualitative method was employed. All women of reproductive age group in Jinka town were participated in the study. For the quantitative survey 800 women in the reproductive age group were participated. The sample size was computed by using single population proportion formula for finite population with 95% confidence level, prevalence of modern contraceptive use in southern nation and nationality people region of 11.4% and marginal error of 2%. Labeling of households in the study area was done prior to the actual study conducted to construct sampling frame and to determine the total number of households found in the study area. The actual respondents were selected by systematic sampling method at household level. Qualitative study participants for focus group discussion were selected by purposive sampling technique.

### Data collection

Data were collected by face to face interview using interviewer administered questionnaire for the quantitative study. The data collectors and supervisors were trained on sampling procedures and interview techniques. The data collectors visited sampled households and made the interview by using structured questionnaire. Four focus group discussions were held among two from male and two from female groups that composed of 10 discussants by using interview guides. While conducting focus group discussion, explanation and elaboration were done and their idea was tape recorded. To maintain the quality of data, training of data collectors and supervisors, pre-tested, regular follow up of data collectors were done during the study period at field. Furthermore, incompleteness and inconsistency of the data were checked, edited and cleaned.

### Statistical analysis

The data were entered to statistical software of Epi Info window version 3.3 and exported into SPSS window version 13.0. Descriptive statistics were computed to determine the frequency of dependent and independent variables. Binary and multiple logistic regressions were done to determine the presence or absence of statistical relationship of the outcome variable and the explanatory variables. Statistical association was declared by using adjusted odd ratio and at p

### Ethical review

The study was ethically approved by the ethical clearance committee of college of health and medical sciences, Addis Ababa University. Permission was obtained from South Omo zone health department and Jinka town. Verbal consent was obtained from participant. Confidentiality and the right of respondents were respected.

## Results

### Sociodemographic and reproductive health history

Seven hundred sixty three women in the reproductive age were participated that made response rate of 94.4%. The majority (89.4%) of participants were in the age group of 15-39 with mean age of 28.3 years. Most 368 (48.2%) of participant were Amhara, Orthodox 469(61.5%), married, 469(61.5%), more than half (58.8%) of them were housewives and two third of them were attend regular education (Table 1). Forty one percent of participants had their first marriage before 18 years, and half of them had given their first birth at age of 18 and 336(44%) of the participants want to have 3-4 children. Three fourth (75.5%) of participants were ever pregnant, 338(44.3%) participants have more than four family size and the average family size of participants was 4.95(+2.2). The single participants want to have their first pregnancy on average age of 25.1(+3.1) years.

**Table 1:** Sociodemographic characteristics of women, Jinka town, South Ethiopia, 2008 (n = 763)

Socio demographic Characteristics	Frequency	Percent (%)
**Age group of participants**		
15-24	245	32.1
25-34	303	39.7
35-49	215	28.2
**Marital Status**		
Married	535	70.1
Single	148	19.4
Divorced/ Separated	38	5.0
Widowed	42	5.5
**Educational Status**		
Illiterate	171	22.4
Read and write	87	11.5
1-12 grade	407	53.3
College/Univ.	98	12.8
**Occupation**		
Student	146	19.1
Gov/NGO	119	15.6
House wife	449	58.8
Others	49	6.5
**Religion**		
Orthodox	469	61.5
Muslim	82	10.7
Protestant	189	24.8
Catholic	15	2.0
Others	8	1.0
**Ethnic Composition**		
Amhara	368	48.2
Ari	132	17.3
Gofa	84	11.1
Wolayta	46	6.1
Besketo	58	7.6
Oromo	24	3.1
Others	51	6.8

### General Awareness on long acting and permanent methods

Eighteen percent of participants have ever heard about long acting and permanent methods (95% CI: 15.4-20.9). Majority, 110(80%) of those with knowledge on long acting and permanent methods were married women. The respondents had information about the importance of long acting and permanent methods such as prevent unwanted pregnancy, used for child spacing, use to limit family size and use to prevent maternal mortality and morbidity74.6%, 77.5%, 63%, 48.6% respectively. Ninety three percent of women were obtain information from health institution about long acting and permanent contraceptive methods. More than three fourth of women have ever heard implant, 55(39.9%) about female sterilization and 53(38.4%) about intra uterine contraceptive device.

Seventy two percent of participants have knowledge about intra uterine contraceptive device. Moreover, 72(52.2%) know its effectiveness, 45(32.6%) know that used for more than ten years. About 113(84.9%) of women have knowledge of implants. Moreover, less than half, 61(44.2%) of the participants know that it is very effective, 76(55.1%) know that it has long-term use. Forty eight percent of women had knowledge on vasectomy. Furthermore, one third of participants know that vasectomy is permanent method and 26 (18.8%) know that it has no effect on sexual performance and sensation. More than two third participants have knowledge on female sterilization.

The awareness level of study subjects were supported by qualitative study. Most discussants expressed their view that there is very low knowledge and use of long acting and permanent methods in their community. A 30 years old female discussant said,*“…. I know long acting and permanent methods since we occasionally got educations on it from health facilities, but the community mostly use depo provera than long acting and permanent methods. If there is use of long acting and permanent methods at all, sure, it is too minimal from the total and I think there is too little knowledge too. Those who live in towns may have the knowledge about depo provera only…”*

From the long acting and permanent methods, which were raised for discussion, majority of those who know, are only familiar with implant. Moreover, some discussants expressed that there is knowledge and use of implants. A participant, (32, F) has said, *“…Mostly, the communities know about Implant. However, I don't think the communities have better knowledge about intra uterine contraceptive device and male sterilization. There is thinking that intra uterine contraceptive device and female sterilization can cause internal problem to the user. I heard that there is a method of family planning by sterilizing male but I don't hear about the one who use male sterilization method in my locality.”*

### Attitudes and intention to use long acting and permanent methods

In general, about 332(43.5%) participants have supportive attitude towards long acting and permanent methods. However, 239(31.5%) participants have poor attitude on long acting and permanent methods. Nearly half, 379(50.3%) of participants used to communicate on long acting and permanent methods s and 374(98.7%) of those who communicate with their partner /friends support use of long acting and permanent methods. In addition, study participants support use of long acting and permanent methods and have an intention or plan to use it 689(90.3%) and 468(68%) respectively. Four hundred seventy one (61.7%) of participants explained that their partners have good attitude or support use of long acting and permanent methods. Furthermore, majority, 605(79.3%) of participants believe that it is the couples responsibility to use contraceptive and most of participants, 747(97.9%) and 744(97.5%), believe that having giant family size poses great problem on economy and maternal health respectively.

Different variables, 90(53%) of illiterate, 51(64.6%) of those who can read and write, 276 (68.3%) of those with modern education, 57(58.2%) of those with college education have intention to use long acting and permanent methods. Moreover, participants who perceive their social status to be very poor, poor, medium and rich have intention to use long acting and permanent methods were 7(58.3%), 111(55.8%), 334(65.4%) and 13(68.4%) respectively (Table 2). Intention of study subjected to uses long acting and permanent fertility control methods also were supported by qualitative. In general, most participants had positive attitude towards using contraceptives. Most participants agreed that the use of contraceptives in general and long acting methods in particular, especially Implant for birth spacing are good to the health of children, mothers and family. Moreover, limiting family size is important for a country and society. One discussant, (30, F) expressed her view saying: *“…It is good if everyone uses family planning methods because of the current difficult living condition. Moreover, mothers suffer much due to birth, can die during birth and also children may die due to frequent birth hence, it is better to use family planning methods. Therefore, limiting the number of children and using family planning is good in my opinion…”*

**Table 2:** Attitudes of women towards long acting and permanent contraceptive methods, Jinka town, South Ethiopia, 2008 (n = 763)

Characteristics(Attitude factors)	Frequency	%
**Support use of LAPMs**		
Yes	689	90.3
No	41	5.4
No idea	16	2.1
Don't concern me	8	1.0
No response	9	1.2
**Communication with friend/Husband**		100.0
Yes	379	49.7
No	375	49.1
No response	9	1.2
**Friend/Husband attitude on LAPMs**		
Support	471	61.7
Against	76	10.0
Neutral	5	0.7
I don't know	133	17.4
Don't concern me	71	9.3
No response	7	0.9
**Responsibility in using LAPMs**		
Wife	61	8.0
Husband	38	5.0
Both	605	79.3
Don't know	13	1.7
Don't concern	40	5.2
No response	6	0.8
**Large family has problem on economy**		
Yes	747	97.9
No	8	1.0
Don't know	3	0.4
No response	5	0.7
**Large family has problem on MCH**		
Yes	744	97.5
No	10	1.3
Don't know	4	0.5
No response	5	0.7
**Intention/plan to use LAPMs**		
Yes	478	62.6
No	208	27.3
No idea	16	2.1
Don't concern me	54	7.1
No response	7	0.9
**Attitude on LAPMs composite measure**		
Good attitude	332	43.5
Poor attitude	239	31.3
Non response	192	25.2

Most participants seriously express that it is very essential to limit family size due to the current harsh living condition. In order to limit family size most participants thought that it is better to use shorter acting and long acting once rather than permanent methods. For their preference, they site different reasons. Participants also suggest that permanent contraceptives should be used by those who want no more children, HIV/AIDS patients, street children and others with no income. A male participant (31, M, protestant) from town said, *“…The permanent methods can cause regret if a long acting and permanent methods user, having one or more children, wants to have additional child when he/she improve economically. Hence, rather than using permanent methods it is better to use methods like depo and intra uterine contraceptive device. In addition, providing education concerning permanent methods is mandatory…”* There was very poor attitude concerning vasectomy by most male participants. One male participant, (54, M), expressed said, *“…If I have vasectomy, I can't have birth. Why should I use this method?! Shouldn't I replace myself?! Nobody should use vasectomy, unless by legal punishment. Previously, there was no issue about male sterilization.’’*

The overall prevalence of contraceptive utilization among participants was about 301(39.5%). Of which majority, 242(31.6%) were users of modern contraceptive other than long acting and permanent methods. Long acting and permanent methods users were 56(7.3%) (95% CI:5.6-9.5%) in married women. During the study period women used long acting methods such as implant and intra uterine contraceptive device were 28 (50%), 7(12.5%) respectively. Among permanent methods, 20(35.7%) of women used female sterilization, and only one study subject used vasectomy. All current users (56) got long acting and permanent methods from government health facilities. There were several reasons for not using long acting and permanent methods. Among those ninety-seven (16.2%) participants were not using it because they are single, 66 (11%) of them want to have child (pregnant) and they have health problems and 65(11%) were due to fear of health effect and others.

### Factors affecting Long Acting and Permanent Methods use

Several socio demographic, reproductive health and other factors like knowledge, attitude were tested for the presence of association with long acting and permanent methods ever use by using binary logistic regression analysis. Variables like age of women, educational status, monthly income, family size, total number of children participants want to have, knowledge on long acting and permanent methods and presence of communication with husband or friends were found to be significantly associated with long acting and permanent methods use. These factors were further analyzed using multiple logistic regressions.

Knowledge on long acting and permanent methods was found to be an important predictor of long acting and permanent methods use (OR 145.6, 95% CI: 29.0-730.2). The very wide confidence interval is due to small sample size. Moreover, people in the age group of 25-34 years (OR 6.5, 95% CI: 1.4-29.5) and 35-49 years (OR 6.2 95% CI:1.3-30.4) were more than six times users of long acting and permanent methods compared to those in the age group of 15-24 (Table 3). Almost all of the focus group discussants raised many factors and misconceptions that hinder use of long acting and permanent methods such as husband disapproval, considering children as assets, fear of sterility lack of knowledge, cultural and religion disapproval and fear of several side effects such as heavy period, slipping out during heavy work in the case of intra uterine contraceptive device.

**Table 3:** Predictors of long acting and permanent contraceptive methods, Jinka town, South Ethiopia, 2008

Variables	LAPMs Ever use		COR(95%CI)	AOR (95%CI)
	Yes	No		
**Age**				
15-24	4	241	1	
25-34	28	275	6.14(2.12-17.73)	6.51(1.44-29.49)*
35-49	26	173	9.06(3.10-26.15)	6.22(1.28-30.36)*
**Education**				
Illiterate	8	132	1	1
Read and write	8	72	2.25(0.81-6.23)	0.57(.09-3.61)
1-12 grade	24	379	1.28(0.56-2.92)	0.40(.09-1.79)
College/Univ	17	81	4.25(1.76-10.26)	0.95(0.16-5.67)
**Monthly income**				
<500 birr	17	321	1	1
500-1000 birr	28	192	2.75(1.469-5.163)	2.05(0.65-6.42)
>1000	9	83	2.05(0.88-4.76)	1.24(0.29-5.42)
**Family size**				
<4 in number	14	305	1	1
> = 4 in number	35	300	2.54(1.34-4.82)	1.20(.44-3.31)
**Total children wanted**				
1-2 child	7	206	1	
3-4	28	305	2.70(1.158-6.301)	0.8(0.23-3.54)
≥5	19	130	4.30(1.76-10.52)	2.294(0.43-12.31)
**Communication with husband/friend**				
No	3	370	1	
Yes	55	324	20.94(6.488-67.56)	1.99(0.45-8.80)
**Knowledge on LAPMs**				
No	5	614	1	1
Yes	53	84	77.48(30.12-199.32)	145.6(29.03-730.2)*

* p-value < 0.05, * indicates those with significant association.

## Discussion

This study had attempted to assess the prevalence of long acting and permanent fertility control methods and associated factors. Approximately four out of every ten women used modern contraceptive. The prevalence of long acting and permanent contraceptive method was 7.3%. About one out of every five participant has ever heard information about one or more of long acting and permanent contraceptive methods. Two third of participants reported to have intention to use long acting and permanent methods. Female sterilization was three percent in which it accounts almost one third of the long acting and permanent contraceptives. Less than one percent of study participants were used intra uterine contraceptive device and vasectomy. Knowledge of contraceptive and age of women have significant association with use of long acting and permanent contraceptive.

About one out of every five participant has ever heard information about one or more of long acting and permanent methods. Knowledge concerning long acting and permanent methods was very low. This finding is similar with a finding from the Ethiopian demographic and health survey 2005. However, this is lower than a study done in Tehuledre woreda, Ethiopia, [6, 7]. This may be due to lack of describing and probing of methods not mentioned spontaneously by participants.

Generally, knowledge concerning long acting and permanent methods was very low. It was also raised by most focused group discussants that there is very low knowledge on long acting and permanent methods probably for there were no routine health education programs on long acting and permanent methods. Moreover, most of those with knowledge on long acting and permanent methods were married and those with some education, which is consistent with other studies [6, 7].

This study indicated that two third of participants had intention to use long acting and permanent methods. This is consistent with a study done in Tehuledre [7]. Moreover, it is in line with Ethiopian demographic and health survey 2005 finding where 42% women want no more children and there was 14% unmet need for limiting [6]. This result is also consistent with a study in Bure; north western Ethiopia found that more than half of women had reported unmet need. Furthermore, in the same study it was found that 43.1% preferred long acting and permanent methods [8]. It is similar with study done in info project revealed that three quarter of women preferred long acting methods and half of women who were not using any contraception but intend to use contraceptive in the future [2]. This is similar with a study done in Gondar found that more than two third of both female and male had positive attitude and intention to use contraceptives [9]. There is also very high intention to use contraceptives and have positive attitude towards in general as suggested by focus group discussants.

This study showed that approximately four out of every ten women used modern contraceptive. Among modern contraceptive users, more than half of them were married couples and prevalence of long acting and permanent method was 7.3%. This study is much higher than a finding from Ethiopian demographic and health survey 2005, findings in different part of the country such as Bure and Gander found that use of intra uterine contraceptive device and Norplant users were less than one percent [6, 8, 9]. These may be due to the recent provision of training and supplies of long acting methods in the area, attitude change of people towards using long acting methods. Two third of participants have been attended modern (1-12 grades) or college education and provision of female sterilization methods to those visiting hospital for delivery. On the other hand, this finding was consistent with study done in Zambia found that long acting contraceptive was eight percent. But this study contradicts with a study done in South Africa showed that almost one in every five women used long acting contraceptive, and this finding is very low when compared with Latin American countries Dominican Republic and India [7, 10, 11].

This study revealed that female sterilization was almost three percent in which it accounts almost one third of the long acting and permanent contraceptives. This finding is relatively higher than some studies in Ethiopia [6]. However, it is very low compared with a study in Tehuledre, Ethiopia found that female sterilization accounts almost half of all long acting and permanent methods. This finding is also similar with a study done in Tunisia, Bangladesh and Jordan [7, 12]. But this study is quite lower than a study done in Asia; Latin America and Caribbean countries found that more than one third of women were user of female sterilization [13]. These different may be due to different socio demographic, cultural and service related factors in different areas.

This study indicated that less than one percent of study participants used intra uterine contraceptive device and vasectomy. This finding is comparable with a study done in African countries [7, 14]. The reasons for low use of intra uterine contraceptive device and vasectomy may be due to lack of knowledge and misconception about long acting and permanent contraceptive methods as raised by discussants. The women who have knowledge on long acting and permanent contraceptives method were more likely used the methods. This is similar with a study done in Butajira, southern Ethiopia showed that women who had high knowledge about long acting and permanent method were more likely to practice long acting and permanent method as compared with those had low knowledge [15]. This study showed that older women were more likely used long acting and permanent contraceptive methods as compared to the younger age women. This is consistent with a study conducted in Batu town central Ethiopia were older age group of women more likely to use long acting and permanent methods [16].

### Strength and limitation of the study

The strength of the study used community based both quantitative and qualitative primary data. But the study design was cross sectional so that cause and effect relationship could not be established. So analytical study design is recommended to establish cause and effect relationship between long acting and permanent methods and associated factors.

## Conclusion

The study had demonstrated that the overall prevalence of long acting and permanent contraceptive method was low. Most of the participants have intention to use long acting and permanent fertility control methods in the future. Knowledge of contraceptive and age of women have significant association with the use of long acting and permanent contraceptive methods. Therefore, extensive health information should be provided through behavioral change communication and information education communication strategy integrated with health extension program.

## References

[1] Guillebaud J (2007). Optimum population thrust, youth quick, population, fertility and environment in the 21st century, UK.

[2] Ruwaida M Salem, Mahua M, Catherine E Richey, Robey B, Blackburn R (2004). The Info project, Center for communication program, population report, the reproductive revolution continues, Johns Hopkins Bloomberg School of public health. Spring.

[3] Central statistical agency (Ethiopia) (2007). Ethiopian population census 2007.

[4] Jelaludin A, Mengistu G (2002). Evaluation of program options’ to meet unmet need for family planning in Ethiopia.

[5] Scholl E, Schueller J, Gashaw M, Wagaw A, WoldeMichael L Assessment of youth reproductive health programs in Ethiopia. http://www.fhi360.org/resource/assessment-youth-reproductive-health-programs-ethiopia.

[6] Central Statistical Agency (Ethiopia) and ORC Macro (2005). http://dhsprogram.com/pubs/pdf/FR179/FR179[23June2011].pdf.

[7] Asnake M, Walie L, Melkamu Y (2007). Improving the range of contraceptive choices in rural Ethiopia, Tehuledre woreda, in South Wollo zone, Amhara Regional State, Ethiopia. Ethiopian Journal of health development..

[8] Alamineh L (2007). Assessment of family planning method mix and effects of lack of preferred contraceptive methods in Bure woreda, West Gojjam zone, Amhara Regional State, Ethiopia.

[9] Kebede Y (2000). Contraceptive prevalence and factors associated with usage of contraceptives around Gondar Town, Ethiopia. J Health Dev..

[10] Population Reports (1900). Approval of voluntary sterilization growing in Islamic countries. http://www.highbeam.com/doc/1G1-11427683.html.

[11] Banerjee B (2004). Socio-economic and cultural determinants on acceptance of permanent methods of contraception, Hooghy district, West Bengal, India. The Journal of family welfare..

[12] Melkamu Y, Betre M (2005). Effects of Contraceptive Shortages and Coping Mechanisms in Selected Regions of Ethiopia: Consortium of reproductive health association.

[13] Eric ES, Jane T Bertrand, Tara MS (2007). Changes in contraceptive method mix in developing countries. International Family Planning Perspectives.

[14] USAID (2006). The Acquire project: Get permanent smile increasing Awareness of Access to, and utilization of vasectomy services in Ghana.

[15] Temesgen A (2007). Assessment of the prevalence and factors influencing the utilization of long acting and permanent contraceptive method in Butajira town, Gurage zone, southern Ethiopia.

[16] Haile A, Fantahun M (2012). Demand for long acting and permanent contraceptive method and associated factors among family planning service users, Batu town, central Ethiopia. Ethiop Med J..

